# Automatic computation of breast cancer biomarkers from multiple $^{18}$ F-FDG PET image segmentation

**DOI:** 10.1038/s41598-026-50945-w

**Published:** 2026-05-18

**Authors:** Tewele W. Tareke, Neree Payan, Alexandre Cochet, Laurent Arnould, Benoît Presles, Jean-Marc Vrigneaud, Fabrice Meriaudeau, Alain Lalande

**Affiliations:** 1 Université Bourgogne Europe, ICMUB UMR 6302, Dijon, 21000 France; 2 Université Bourgogne Europe, Centre de Lutte contre le Cancer G.-F. Leclerc, Département de Médecine Nucléaire, CNRS, ICMUB UMR 6302, Dijon, 21000 France; 3 Université Bourgogne Europe, Centre de Lutte contre le Cancer G.-F. Leclerc, Département de Biologie et de Pathologie des Tumeurs, Dijon, 21000 France; 4 Université Bourgogne Europe, CHU Dijon Bourgogne, Pôle Imagerie, CNRS, ICMUB UMR 6302, Dijon, 21000 France

**Keywords:** Breast cancer, $^{18}$F-FDG PET, Biomarkers, Segmentation, Deep learning, Quality control, Neoadjuvant chemotherapy, Biomarkers, Cancer, Computational biology and bioinformatics, Oncology

## Abstract

Neoadjuvant chemotherapy is a standard clinical practice for tumor downsizing in breast cancer, with $^{18}$F-FDG Positron Emission Tomography (PET) being an essential tool for predicting complete pathological response and monitoring treatment. Our work aims to leverage PET imaging for automated segmentation of breast lesions and biomarker analysis before and after the first course of chemotherapy. We developed a system to segment primary tumor regions and extract biomarkers reflecting tumor evolution. A total of 243 baseline and 180 follow-up $^{18}$F-FDG PET scans were acquired. Ground truth annotations were generated semi-automatically for all baseline scans and manually for 12 test cases. A deep learning-based segmentation method was developed and evaluated using various architectures. The optimal baseline model, trained on baseline exams, was fine-tuned on 15 follow-up scans using active learning to segment tumors in follow-up exams. The pipeline extracts maximum standardized uptake value (SUV$_{max}$), metabolic tumor volume (MTV), and total lesion glycolysis (TLG) to assess tumor response. Quality control procedures were used to exclude outliers. Among the tested approaches, nnUNet achieved the best tumor segmentation on PET baseline scans, with a Dice similarity coefficient of $0.89 \pm 0.04$ and a Hausdorff distance of $3.52 \pm 0.76$ mm on the test set. After fine-tuning, performance on follow-up exams reached a Dice similarity coefficient of $0.78 \pm 0.03$ and Hausdorff distance of $4.95 \pm 0.12$ mm. Biomarker analysis showed strong correlations between manual and automatic segmentations. The average $\Delta SUV_{max}$, $\Delta MTV$, and $\Delta TLG$ between 12 baseline and follow-up scans used for testing were $-6.02 \pm 1.55$ (p=0.002), $-9.30 \pm 2.33$ cm$^3$ (p=0.007), and $-14.09 \pm 6.33$ cm$^3$ (p=0.010), respectively. The proposed method provides an effective automated system for breast tumor segmentation from $^{18}$F-FDG PET. Thanks to biomarkers extracted from the automatic segmentations on both baseline and follow-up exams, our method enables the automatic assessment of cancer progression.

## Introduction

Breast cancer is one of the most widespread and concerning forms of cancer worldwide, particularly affecting women. Lichtenstein et al. have pinpointed specific genetic variations linked to higher breast cancer risk, providing valuable insights into its causes^1^. Neoadjuvant chemotherapy (NAC) was initially introduced as a treatment strategy for managing inflammatory or locally advanced breast cancers that are deemed inoperable at diagnosis^2^. This approach allows for the reduction of tumor size or stage before surgical intervention and improving overall treatment outcomes. Then NAC is the first-line treatment for patients with inoperable or locally advanced breast cancer^3^. Minckwitz et al.^4^ have made the link between pathological complete response (pCR) and prognosis following NAC across various intrinsic breast cancer subtypes.

Positron Emission Tomography (PET) imaging is widely used to evaluate the spread of disease during diagnosis and treatment. It does so by analyzing the functional activity of cancerous cells within tissues. PET imaging is a crucial asset in the NAC framework, enabling early and precise assessment of treatment response in breast cancer patients^5–7^. Berriolo-Riedinger et al.^8^ assessed the predictive value of reduced $^{18}$F-FDG uptake in breast cancer patients undergoing NAC in relation to achieving a pCR. Cochet et al.^9^ evaluated the impact of $^{18}$F-FDG Positron Emission Tomography/Computed Tomography (PET/CT) on clinical management and its prognostic value in the initial staging of newly diagnosed large breast cancer. Another prospective study has evaluated the relationship between tumor blood flow, glucose metabolism (assessed by $^{18}$F-FDG PET), and proliferation and endothelial pathological markers in newly diagnosed breast cancer^10^. Biomarkers such as maximum standardized uptake value (SUV$_{max}$), metabolic tumor volume (MTV), and total lesion glycolysis (TLG) derived from PET imaging are essential for providing insights into tumor metabolism and size^11–14^. Humbert et al. analyzed the evolution of biomarkers, such as $\hbox {SUV}_{{max}}$, to evaluate their predictive value in breast cancer treatment and their impact on clinical decision-making^6^. Similarly, a prospective study by Schwarz-Dose et al. found that biomarker measurements were significant in predicting pathological response after the first treatment cycle^15^. Follow-up $^{18}$F-FDG PET scans ($\hbox {PET}_{{Fu}}$) is a widely evaluated exam for distinguishing metabolic responders from non-responders in interim or post-treatment PET scans^7^. This review evaluated treatment response by calculating the percentage reduction in $\hbox {SUV}_{{max}}$ observed on follow-up PET scans after NAC, compared to baseline scans. This analysis was assessed both in $\hbox {SUV}_{{max}}$ changes and the pathological complete response. Gallivanone et al.^16^ have demonstrated that biomarkers such as $\hbox {SUV}_{{max}}$, TLG, and textural features extracted from pre-treatment $^{18}$F-FDG PET and magnetic resonance imaging (MRI) are able to define patient prognosis and predict the response to NAC in breast cancer.

Accurate breast lesion segmentation on PET scans plays a vital role in improving the precision of biomarker analysis. By separating the tumor from surrounding tissues, segmentation enhances the reliability of predictive evaluations. This process supports personalized treatment planning and the monitoring of patient progress. However, segmenting functional volumes in PET images presents a significant challenge due to several factors. Manual delineation, while an option, is subjective, laborious, and time-consuming in medical imaging. Before 2007, the majority of breast cancer segmentation techniques consisted of using a binary threshold from PET image intensities. This thresholding often relied on methods like selecting a percentage of the $\hbox {SUV}_{{max}}$ value, setting an absolute threshold for maximum concentration of the tracer within a region of interest, or employing adaptive thresholding techniques^13,17–21^. Advanced image processing and computer-aided methods have emerged for processing breast cancer images^22–26^. However, their effectiveness may still be limited by factors such as tumor heterogeneity and low signal intensity, such as those affected by NAC^27^.

Recent advances in molecular imaging, particularly with PET/CT, have transitioned from purely diagnostic roles to serving as critical tools in personalized management and clinical decision-making. Alonzo et al.^28^ conducted a systematic review highlighting the significant impact of molecular imaging in identifying tumors of unknown primary origin and suspected neuroendocrine tumors (NETs). Their findings emphasize that while PET/CT is established, its application in emerging areas such as neuroendocrine neoplasms provides a high diagnostic yield, often altering the therapeutic pathway by localizing primary lesions that conventional imaging fails to detect. This clinical utility is further enhanced when integrated into predictive systems. Canfora et al.^29^ showed the potential of classifying preoperative grading in cancer using a predictive system based on radiomic features. Their work suggests that the high-throughput extraction of quantitative data from standard images can categorize tumor aggressiveness, bridging the gap between raw imaging and clinical prognosis.

Our study focuses on 3D PET images obtained through a specific protocol for women undergoing treatment for breast cancer^13^. In summary, a total of 243 PET scans at baseline and 180 scans at follow-up were included in our study. Baseline PET/CT ($\hbox {PET}_{{Bl}}$) scans were captured before the initiation of NAC or during the early stages of cancer, prior to any treatment. Follow-up PET/CT ($\hbox {PET}_{{Fu}}$) scans were acquired after the completion of the first course of NAC. The aim of our study was to develop an automated algorithm for segmenting breast cancer lesions on PET scans. The model was trained and validated using $\hbox {PET}_{{Bl}}$ scans and applied also on $\hbox {PET}_{{Fu}}$ scans. The objective was to develop a model capable of accurately segmenting $\hbox {PET}_{{Fu}}$ scans using trained architecture from $\hbox {PET}_{{Bl}}$ scans, while also extracting imaging biomarkers. To achieve this, UNet-based convolutional neural network architectures were employed^30–32^ specifically designed for image segmentation tasks. For $\hbox {PET}_{{Fu}}$ scans, the model developed on $\hbox {PET}_{{Bl}}$ scans was fine-tuned using a sample of $\hbox {PET}_{{Fu}}$ scans. Quality control tools were integrated into the system to ensure robustness and reliability. Moreover, our automatic segmentation method was compared with manual drawing considering inter-observer variability. Finally, three key imaging biomarkers ($\hbox {SUV}_{{max}}$, MTV, and TLG) were extracted in $\hbox {PET}_{{Bl}}$ and $\hbox {PET}_{{Fu}}$ examinations.

## Material and methods

### Data acquisition

**Fig. 1:**
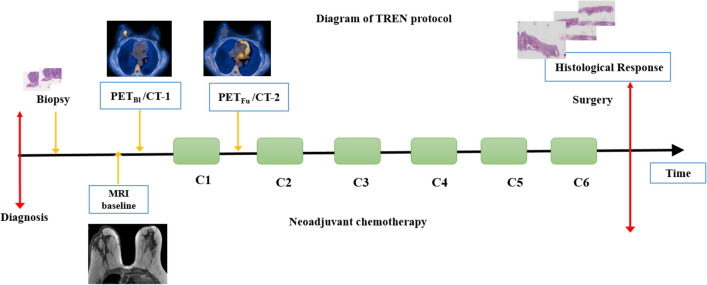
Diagram of our protocol. The data from the $\hbox {PET}_{{Bl}}$ scan performed before the start of treatment are used to quantify the initial metabolism of the tumor. The data from the $\hbox {PET}_{{Fu}}$ scan performed after the first course of chemotherapy are used to assess residual metabolism of the tumor and the metabolic response compared to the results of $\hbox {PET}_{{Bl}}$. Magnetic Resonance Imaging (MRI) exam, which is an addition to the main protocol, was frequently performed at baseline step.

The data were collected from 254 patients enrolled in a clinical trial called TREN protocol (Recorded on ClinicalTrials.gov identifier: NCT02386709)^13^. The institutional review board approved this prospective study as part of standard care. This protocol aims to evaluate early metabolic and perfusion changes in invasive breast cancer patients undergoing NAC, with the objective of identifying predictive markers of tumor response and prognostic factors for recurrence risk^14^. Successful identification of these factors allows for early adaptation of chemotherapy regimens based on marker evaluation. An overview of the protocol is illustrated in Fig. 1.

This protocol involves administering treatment before the surgical resection, aiming to shrink tumors. Inclusion criteria involve women aged 18 or older with newly diagnosed breast cancer and a tumor diameter larger than 2 cm, eligible for neoadjuvant treatment (stage II or III). Exclusion criteria cover inflammatory tumors, distant metastasis, contraindications to treatment or surgery, pregnancy, high blood glucose level (>9 mmol/l), psychiatric illness impairing study comprehension, and unwillingness for repeated imaging. The protocol is a single-center, prospective, observational, non-randomized study offered to eligible patients who consent to participation^13^. The treatment protocol consists of several courses of NAC (Fig. 1). Once the inclusion and exclusion criteria were applied to the registered patients, 243 patients were retained for our study at the baseline level (Table 1). After the first course of NAC, the number of patients decreases (missed appointment or issues during the PET scan planning). A total of 243 $\hbox {PET}_{{Bl}}$, and 180 $\hbox {PET}_{{Fu}}$ scans were therefore considered for our study. All scans were acquired using a Gemini TruFlight PET/CT scanner (Philips Medical Systems, Eindhoven, The Netherlands), with an axial field of view of 18 cm.

**Fig. 2:**
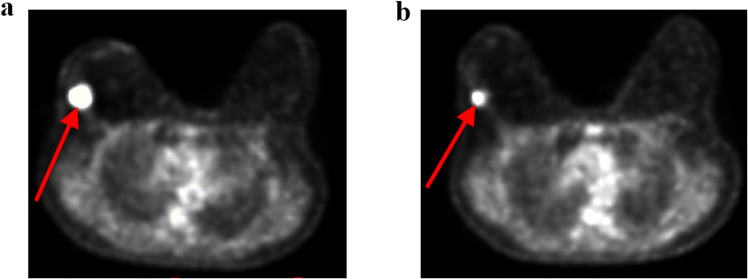
Axial PET images of breast tumor **a** before and **b** after first cycle of NAC for the same patient. The red arrow indicates the tumor lesion.

An automatic PET infusion system (Bayer Medical Care Inc., Indianola, PA, USA) was used to inject a bolus of 3MBq/kg of $^{18}$F-Fluorodeoxyglucose (FDG). The images were acquired in a prone position with a two-step PET/CT scan restricted on the chest. All PET images were reconstructed using a 3D ordered-subset expectation maximization (OSEM) time-of-flight algorithm (3 iterations, 33 subsets) with a 144 $\times$ 144 matrix and 4 mm isotropic voxel size. Emission data were corrected for random coincidences, decay, dead time, scattering and attenuation. Figure 2 shows an example of PET images before and after a first course of NAC from a scan acquired in a prone position and centered on the breast. Among the 243 baseline scans, 231 tumors had already been delineated using a semi-automatic, contrast-based segmentation method^19^. In our study, this delineation method served as the ground truth for training the deep learning models. However, this contrast-dependent approach has been shown to be inadequate for images with low contrast, making it unsuitable for accurately localizing tumors in post-NAC images. Manual segmentation was performed fully manually by experts without the help of any fixed or adaptive SUV thresholding (e.g., SUV 2.5 or percentage of $\hbox {SUV}_{{max}}$). PET tumor volumes were delineated slice by slice based solely on visual assessment of pathological FDG uptake. CT images were used only for anatomical reference to assist localization and boundary identification. In total, 39 PET scans were manually annotated to serve as ground truth, comprising 12 baseline scans and 27 post-treatment follow-up scans (Table 1).

**Table 1:** The distribution of the dataset. $\hbox {PET}_{{Bl}}$; Baseline exam before NAC, $\hbox {PET}_{{FU}}$; Follow-up exam after the first course of NAC, Semi-GT; Semi-annotated ground truth, Ex-GT; Expert-annotated ground truth.

Dataset	Total number	Semi-GT	Ex-GT	Training-validation	Fine tuning	Testing
$\hbox {PET}_{{Bl}}$ exams	243	231	12	231	–	12
$\hbox {PET}_{{FU}}$ exams	180	–	27	–	15	12
Total exams	423	231	39	231	15	24

**Fig. 3:**
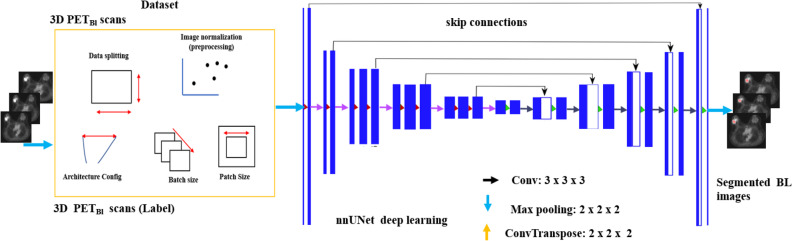
Pipeline of the segmentation of the tumor on $\hbox {PET}_{{Bl}}$ scans. On the left the input data are organized and cleaned before being fed into the network for the segmentation of $\hbox {PET}_{{Bl}}$ scans alongside the ground truth.

**Fig. 4:**
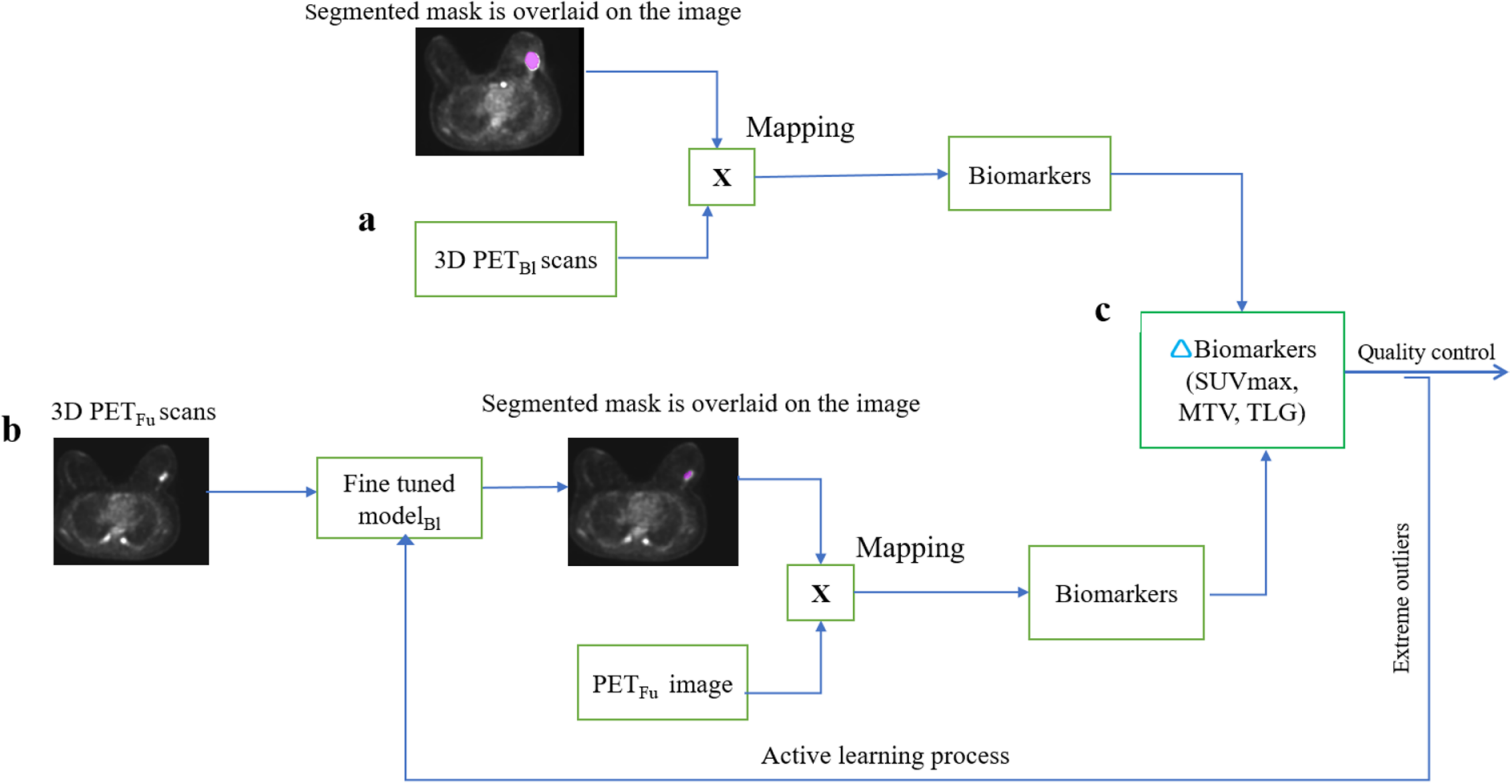
Pipeline for the management of the $\hbox {PET}_{{Fu}}$ scans and the biomarker extraction: **a** Extraction of biomarkers using the segmented mask at the baseline level. **b** Segmentation of the $\hbox {PET}_{{Fu}}$ scans using the fine-tuned baseline model ($\hbox {model}_{{BL}}$). **c** Calculation of changes in the biomarkers between $\hbox {PET}_{{Fu}}$ and $\hbox {PET}_{{Bl}}$, such as $\hbox {SUV}_{{max}}$, to observe the impact of NAC. Active learning process is a process in which outliers identified in the $\hbox {PET}_{{Fu}}$ segmentation by the quality control system are then manually labeled to further refine the model. The term “Mapping” corresponds to the extraction of the biomarkers from the region of interest associated to a segmented mask.

### Deep learning models and data annotation

Our research comprises two main phases. The first phase focuses on segmentation tasks using deep learning techniques applied to $\hbox {PET}_{{Bl}}$ scans, as depicted in Fig. 3. In the second phase, the model is fine-tuned using $\hbox {PET}_{{Fu}}$ scans to segment lesions in the $\hbox {PET}_{{Fu}}$ scans. Our approach is grounded in the robust UNet architecture, widely recognized for image segmentation in medical imaging task^30,33^.

A benchmark of classical deep learning architectures for medical image segmentation has been explored in this work, namely the 2D UNet, 3D UNet and nnUNet. The nnUNet^32^ was implemented using its 3D full-resolution configuration, which is designed to automatically adapt its architecture to the specific properties of the dataset. Given the moderate size of the input volumes, a standardized patch size of $64 \times 64 \times 64$ was utilized to ensure a consistent field of view and sufficient spatial context for lesion identification. The framework employed an ensemble loss function combining Dice and Cross-Entropy to optimize both regional overlap and voxel-wise classification. Training was conducted over 350 epochs using stochastic gradient descent with an initial learning rate of 0.001. To maximize generalization, data augmentation was performed, including random rotations ($\pm 30^{\circ }$), scaling (0.7–1.4), and gamma correction, which simulates the intensity variations commonly found in PET/CT acquisitions. The 3D UNet^31^ was implemented via the MONAI framework, providing a standardized baseline. This architecture utilized five resolution levels with feature channels increasing from 16 to 256, alongside two residual units per level to facilitate gradient flow. Like the nnUNet, this model operated on a $64 \times 64 \times 64$ patch size to maintain consistency across 3D benchmarks. The network used parametric ReLU activation functions and instance normalization to handle the high dynamic range of metabolic imaging data. Optimization was handled by the Adam optimizer with a learning rate of $1 \times 10^{-4}$. Due to the high memory demands of 3D convolutions, early stopping was carefully monitored on the validation set to prevent overfitting while ensuring the model captured the three-dimensional morphology of the tumors. In contrast to the 3D models, the 2D UNet^30^ processed the data on a slice-by-slice basis using axial inputs of $144 \times 144$. This model followed a classical encoder-decoder path with four downsampling stages, where the number of filters doubled at each level to a maximum of 512 at the bottleneck. While it lacks the inter-slice context of the 3D models, the 2D approach benefits from a higher batch size (16 slices) and a more efficient training routine using the Adam optimizer. During the inference phase, the 2D predictions were stacked to reconstruct the 3D volume. The nnUNet architecture enhances the traditional UNet by introducing a flexible, user-friendly framework with automated hyperparameter tuning to improve segmentation performance. Normalization and voxel space alignment of the volumes were implemented for all volumes as pre-processing steps to ensure that the data fed into the network is consistent and optimized for effective learning.

Each 3D $\hbox {PET}_{{Bl}}$ scan has dimensions of 144 x 144 pixels with a depth of 66 slices. We used $\hbox {PET}_{{Bl}}$ scans to develop our proposed deep learning model, considering the semi-annotated 3D $\hbox {PET}_{{Bl}}$ scans^19^ as the ground truth for training and validation. After selecting the best deep learning segmentation method, a fine-tunning approach was implemented. For that, 15 out of the 180 $\hbox {PET}_{{Fu}}$ scans were manually segmented by an expert and used to fine-tune the baseline model. Due to the high cost and time-consuming nature of medical image annotation, we limited the fine-tuning process to 15 follow-up cases. These cases were strategically selected to refine the baseline-trained models while keeping the annotation burden manageable. The primary objective of this fine-tuning strategy was to adapt the model to $\hbox {PET}_{{Fu}}$ scans by leveraging knowledge learned from the baseline model while incorporating specific $\hbox {PET}_{{Fu}}$ characteristics. These 15 $\hbox {PET}_{{Fu}}$ exams were selected on the basis of a quality control process, as explained later in section 2.4. Then, to assess the model’s robustness on unseen data, the 12 reserved $\hbox {PET}_{{Bl}}$ scans and the corresponding 12 $\hbox {PET}_{{Fu}}$ scans were used to test our model and assess its generalization performances. The pipelines are illustrated in Figs. 3 and 4.

### Loss functions

The sum of weighted loss functions, each weighted by a certain factor^34^, was employed. This method enabled the model to handle the imbalance nature of the dataset, enhancing overall segmentation performance. The combined weighted Focal Tversky Loss (FTL)^35^ and Binary Cross-Entropy (BCE) loss functions^36^ was implemented, as it provides a more optimal solution compared to using BCE or FTL individually. The integration of FTL within the network effectively tackled challenges stemming from imbalanced and limited dataset. This is achieved by assigning higher weights to minority classes, improving the classification of rare instances and reducing the effect of class imbalance. The Tversky Index (TI) is defined as:

$$TI = \frac{{\text {TP}}}{{\text {TP} + \alpha \cdot \text {FN} + \beta \cdot \text {FP}}}$$

where TP is the true positive rate, FP the false positive rate, FN the false negative rate, $\alpha$ and $\beta$ control the magnitude of penalties for FPs and FNs, respectively. As TI increases, the Tversky loss function $(1 - TI)$ converges. The main difference between Tversky loss and FTL is the introduction of the focusing parameter $\gamma$ in the latter. This parameter allows the FTL to concentrate more on hard-to-classify cases (i.e. where there is hard-to-segment regions), whereas the standard Tversky loss treats all errors equally (depending on $\alpha$ and $\beta$). The focal Tversky loss is defined as:

$$\text {FTL} = (1 - TI)^\gamma$$

The $\gamma$ parameter controls the level of focusing on hard-to-segment regions. In another hand, Binary Cross-Entropy (BCE) is a loss function used to measure the difference between predicted probabilities and actual binary labels in binary segmentation tasks. BCE loss treats each class equally in terms of penalization, meaning that it assigns the same weight to each class during optimization, regardless of the class distribution in the dataset. The combined loss functions is defined as:

$$\text {Loss}_{\text {weighed}} = \epsilon \times \text {Loss}_{\text {FTL}} + (1 - \epsilon ) \times \text {Loss}_{\text {BCE}}(y, \hat{y})$$

where:

$\hbox {Loss}_{\text {FTL}}$ is the FTL function, $\hbox {Loss}_{\text {BCE}}$ is the BCE loss function with *y* the true value and ($\hat{y}$) the predicted value, $\epsilon$ is the weight parameter representing the relative importance of the FTL compared to the BCE loss. It ranges between 0 and 1, where $\epsilon = 0$ gives full weight to the BCE loss and $\epsilon = 1$ gives full weight to the FTL.

### Quality control

**Fig. 5:**
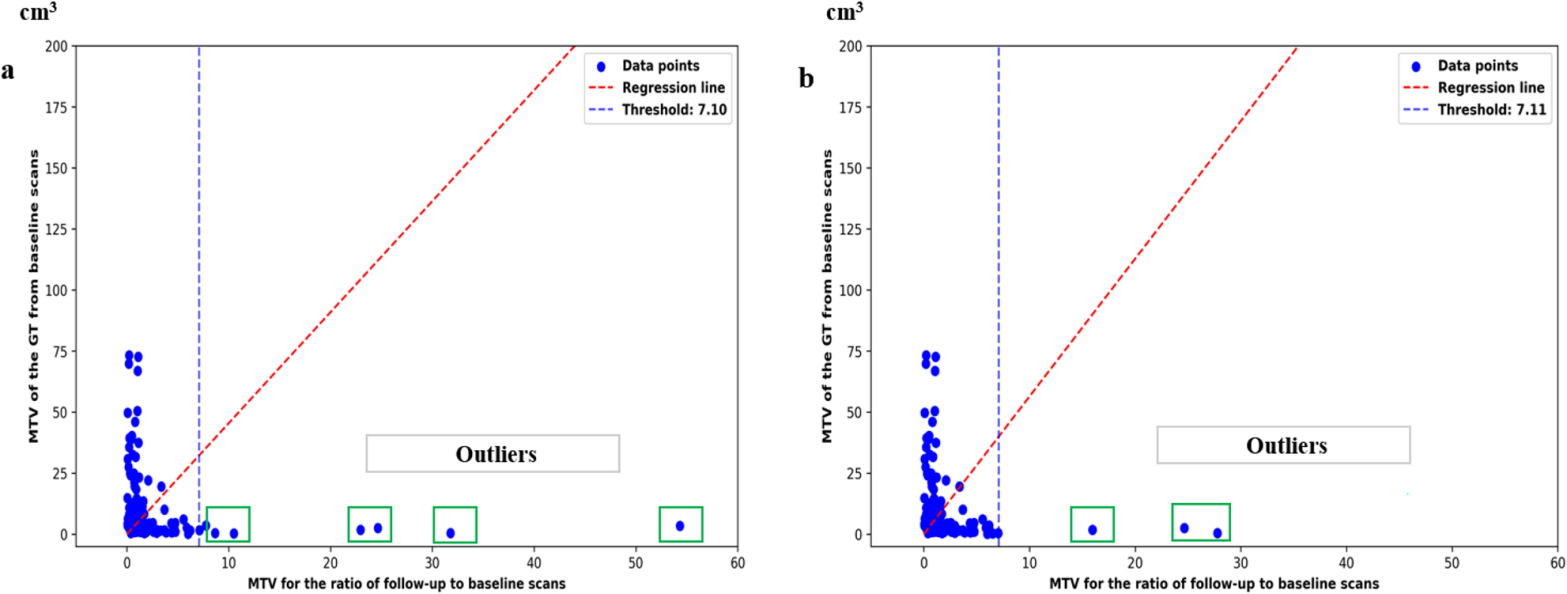
The distribution of MTV in $\hbox {PET}_{{Bl}}$ scans, calculated from labels, is compared to the MTV ratio between $\hbox {PET}_{{Fu}}$ and $\hbox {PET}_{{Bl}}$ cases **a** before and **b** after fine-tuning. The data points within the green rectangles are considered as outliers. The blue perpendicular dotted line to the x-axis represents the threshold. The linear regression line in **a** and **b** represents the linear relationship between the MTV of the GT from baseline scans and the MTV ratio of follow-up to baseline scans, defined by the equation MTV GT = $\alpha$ $\times$ MTV ratio, where $\alpha$ is a constant scalar derived from the mean values.

A quality control step was integrated into the pipeline to verify segmentation accuracy and identify potential outliers. Indeed, we observed a few outliers in the changes of SUV$_{max}$, TLG, and MTV parameters, likely due to unusually large tumor sizes for the predicted masks. An outlier is defined as a data point that lies beyond a specified threshold value within the dataset (example in Fig. 5 for the MTV calculation). The purpose of this quality control approach was to first identify outliers, perform manual segmentation by experts on the corresponding scans, and finally refine the model using these new cases. This quality control step was incorporated as an active learning mechanism to enhance model performance with less labeled examples, which is especially valuable in scenarios where labeling is costly or time-intensive. The quality control process consisted of two steps. First, we assessed whether the segmented masks from $\hbox {PET}_{{Bl}}$ and $\hbox {PET}_{{Fu}}$ scans for a specific patient were localized roughly in the same area. The segmented images from $\hbox {PET}_{{Bl}}$ and $\hbox {PET}_{{Fu}}$ are binarised, with pixel values of 0 for the background and 1 for the cancer region. To objectively track whether the cancer region roughly remains in the same anatomical location over time, we divide each image of the stack of images into four quadrants. The center of mass of the tumor region is considered as the centroid of the region of interest. If centroids from $\hbox {PET}_{{Bl}}$ and $\hbox {PET}_{{Fu}}$ fall within the same quadrant, the segmentation is considered as possible, otherwise, it is flagged as a potential error in the automatic segmentation. Secondly, we calculated the ratio of follow-up to baseline MTV for each patient. An automatic, data-driven threshold was then applied to detect abnormal variations in MTV, calculated from the segmented regions before and after a first cycle of NAC. The threshold value, determined by analyzing the relationship between the MTV extracted from baseline-labeled scans and the ratio of segmented follow-up to baseline tumor volumes across 180 scans, was set at 7.10 or higher. This threshold corresponds to the reciprocal of the mean MTV ratio. In practice, fifteen follow-up cases, including six outliers, were extracted and manually segmented by an expert. These cases were then used to fine-tune the model within the active learning pipeline. Consequently, ratio values exceeding or equal to the threshold of 7.11 were identified as outliers after fine-tuning.

### Inter-observer variability

Inter-observer variability in image segmentation refers to the differences in results obtained when different individuals (observers or annotators) segment the same images. These discrepancies can arise due to variations in the observers’ interpretations, expertise, or approaches. In this study, we aimed to assess the consistency and agreement between two experienced experts, both of whom have over five years of experience in PET imaging of breast cancer. We used 12 test cases at both baseline and follow-up levels to evaluate inter-observer variability. The manual annotations provided by Expert-I were considered as the reference or “ground truth” (GT) for this evaluation. Expert-II, the second observer, blindly performed manual segmentation on the same PET scans. The similarity of these two segmentations allows the assessment of the inter-observer variability. The performance of the automatic segmentation was then compared with the inter-observer variability.

### Evaluation metrics

The evaluation of our model’s performance involves the use of several metrics. The Dice similarity coefficient (DSC) is used for evaluating the similarity between the predicted and ground truth segmentation masks. It measures the overlap between the two, providing a value between 0 and 1, where a higher value indicates better agreement^37^. The intersection over Union (IoU), also known as the Jaccard Index, measures the overlap between the predicted segmentation and the ground truth, relative to their union^37^. It ranges from 0 (no overlap) to 1 (perfect overlap). The sensitivity (True Positive Rate) is used to identify positive instances. The values of sensitivity range from 0 to 1, where 0 indicates that the model failed to identify any true positives, and 1 indicates that the model correctly identified all true positives. The hausdorff distance (HD) quantifies the maximum distance between the predicted and actual segmentation boundaries (the smaller the better)^38^. To evaluate inter-observer variability, we compared the overlap between lesions manually segmented by the two experts using the DSC and HD metrics.

Three biomarkers (the $\hbox {SUV}_{{max}}$, the MTV, and the TLG) were automatically extracted from the fully-automated and the manual segmented regions at both baseline and follow-up stages from the 12 patients used to test the models. The $\hbox {SUV}_{{max}}$ reveals the tumor’s highest metabolic activity for one voxel. Meanwhile, the MTV assesses the tumor’s overall metabolic volume, providing information on disease extent. The TLG combines metabolic activity and volume. The linear relationship between manually and automatically extracted biomarkers using the Pearson correlation coefficient was measured^39^. A paired t-test was conducted to evaluate differences in biomarkers between patients at two different time points (baseline vs. follow-up), as our data follow a normal distribution according to the Shapiro-Wilk test. A p-value of less than 0.05 was considered as significant. Box plots were used to depict the distribution of biomarkers before and after one course of NAC. The whiskers on the box plots represent the spread of data points outside the upper (third) and lower (first) quartiles. Pearson’s correlation coefficient and Bland–Altman analysis^40^ were used to evaluate the correlation and the agreement between biomarkers extracted by the experts and those generated by the developed system.

## Results

### Hyperparameter setting

All experiments were conducted and validated using the 243 $\hbox {PET}_{{Bl}}$ exams mentioned in the Table 1. The effect of varying epsilon values on the combined loss function was studied within the range [0,1]. During the hyperparameter tuning of epsilon, the values of $\alpha$, $\beta$, and $\gamma$ were held constant throughout the experiments and fixed at 0.5, 0.5, and 1.0, respectively. The Table 2 shows that assigning a weighting factor of 0.7 to the FTL and 0.3 to the BCE loss yields optimal segmentation results. We have considered different configurations of $\alpha$, $\beta$, and $\gamma$, along with $\epsilon$, to find the optimal values and improve the segmentation result. The model performed best with $\alpha = 0.7$, $\beta = 0.3$, and $\gamma = 1.5$ (Table 3).

**Table 2:** Comparison of epsilon values in the weighted sum of loss functions on the performance of the UNet-based deep learning model for the segmentation of the tumor on $\hbox {PET}_{{Bl}}$. DSC; Dice Similarity Coefficient, HD; Hausdorff Distance. Best results in bold.

$\epsilon$ values	DSC	$\hbox {HD}_{{mm}}$
0.0	$0.81 \pm 0.06$	$4.81 \pm 1.03$
0.2	$0.83 \pm 0.04$	$7.01 \pm 0.03$
0.3	$0.85 \pm 0.02$	$3.44 \pm 0.97$
0.4	$0.79 \pm 0.09$	$6.00 \pm 2.21$
0.5	$0.86 \pm 0.05$	$4.33 \pm 1.23$
0.6	$0.86 \pm 0.01$	$5.05 \pm 1.77$
**0.7**	$\mathbf { 0.89 \pm 0.04}$	$\mathbf {3.52 \pm 0.76 }$
0.9	$0.87 \pm 0.03$	$3.92 \pm 1.06$
1.0	$0.87 \pm 0.08$	$4.26 \pm 1.06$

### Segmentation results

**Fig. 6:**
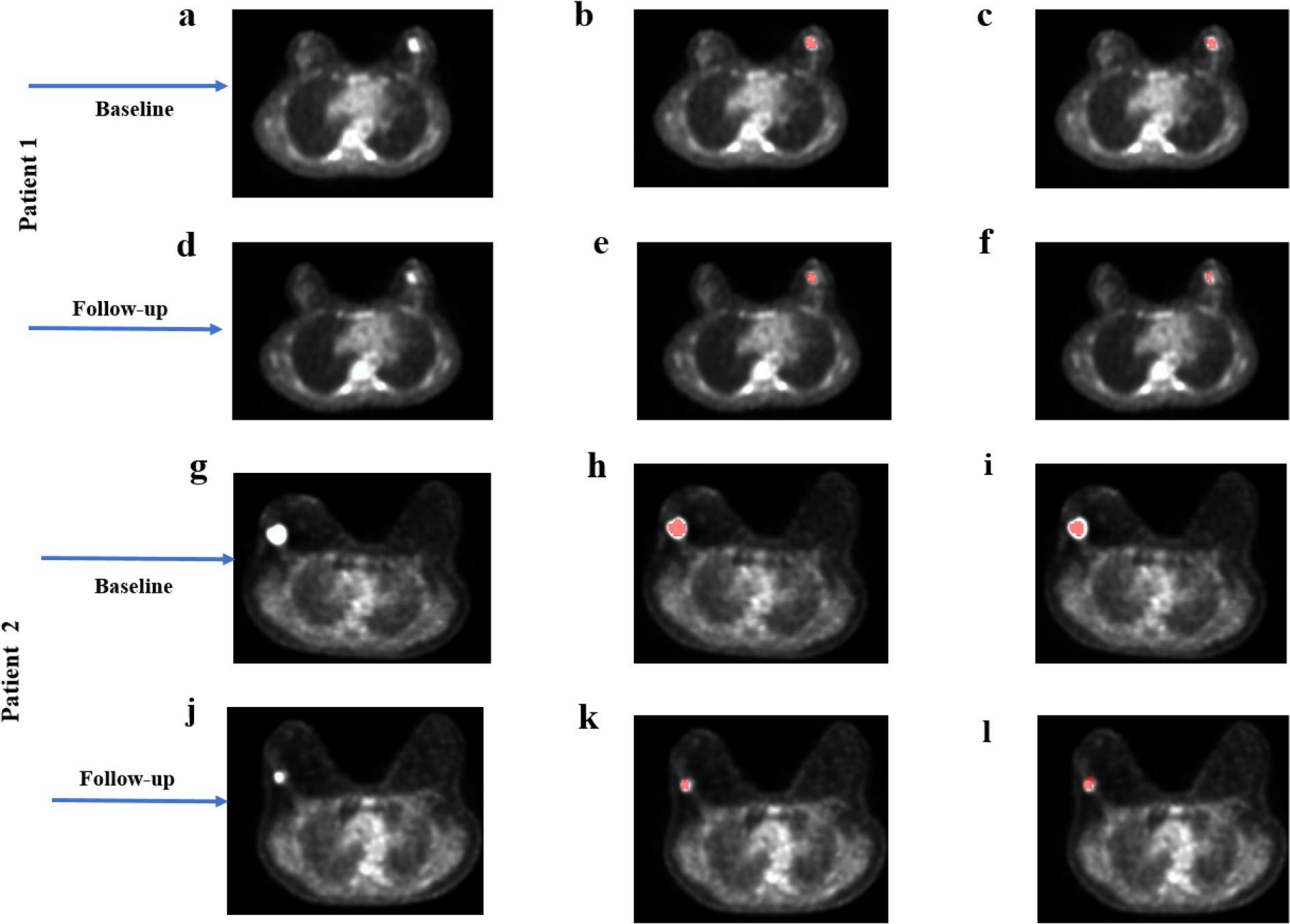
Segmentation results for two patients at both baseline and follow-up stages. For the patient 1, **a** and **d** represent the PET images, **b** and **e** display the corresponding ground truths in red overlaid on the images (**a**) and (**d**) respectively. **c** and **f** show the automatically segmented masks in red for images (**a**) and ( **d**) respectively. Moving on to the patient 2, **g** and **j** depict the PET images, **h** and **k** display the corresponding ground truth in red overlaid on the images (**g**) and (**j**) respectively, and **i** and **l** show the automatically segmented masks in red for images (**g**) and (**j**) respectively.

Among the three UNet-based deep learning architectures evaluated, the nnUNet model was chosen for its superior performance with an average DSC of $0.89 \pm 0.04$ for the best configuration using $\hbox {PET}_{{Bl}}$ dataset (Table 3). Figure 6 shows an example of lesion segmentation for two patients, each including $\hbox {PET}_{{Bl}}$ and $\hbox {PET}_{{Fu}}$ acquisition. The inter-observer variability study between experts for manual segmentation showed a DSC score of $0.92 \pm 0.02$ and HD of $4.02 \pm 0.21$ (Table 4) on $\hbox {PET}_{{Bl}}$ scans. The automatic segmentation achieved an average DSC of $0.89 \pm 0.04$ and HD of $3.52 \pm 0.76$ on the same $\hbox {PET}_{{Bl}}$ scans. For $\hbox {PET}_{{Fu}}$ scans, the inter-observer variability study showed a DSC score of $0.88 \pm 0.01$ and HD of $3.97 \pm 1.90$ (Table 4). The automatic segmentation, on $\hbox {PET}_{{Fu}}$ cases, achieved an average DSC of $0.78 \pm 0.03$ and HD of $4.95 \pm 0.12$. These results indicates that the model’s performance falls within the range of inter-observer variability, highlighting its consistency in replicating expert-level segmentation accuracy. Moreover, the automatic method exhibited a lower HD$_{mm}$ of 3.52 ± 0.76, compared to 4.47 ± 0.12 for the semi-automatic approach. Table 5 presents the baseline model’s performance on the 12 $\hbox {PET}_{{Fu}}$ scans and compares the effectiveness of different adaptation strategies for segmenting breast tumors. The processes of fine-tuning and active learning led to a slight improvement in the segmentation results (average DSC of $0.78 \pm 0.03$). The output in Fig. 12 demonstrates the quality control step, utilizing the center of mass to assess the overlay of the volume of interest between $\hbox {PET}_{{Bl}}$ and $\hbox {PET}_{{FU}}$ scans. For the first example, the location of the ROIs and the ratio of the MTV between $\hbox {PET}_{{FU}}$ and $\hbox {PET}_{{Bl}}$ scans ($\frac{6}{5}$) were determined to be acceptable. For the second examples, although the ROIs were in the same quadrant, the ratio of MTV between $\hbox {PET}_{{FU}}$ and $\hbox {PET}_{{Bl}}$ scans was $\frac{27}{1}$, which was identified as an outlier.

**Table 3:** Comparison of baseline model performance according on the 12 patients to the segmentation results with deep learning architectures and hyperparameter tuning techniques. Best results in bold.

Methods	Parameters	DSC	IoU	Sensitivity	HD$_{mm}$
2D-UNet	$\alpha =0.3, \ \beta =0.7,\ \gamma =1$	$0.71\pm 0.07$	$0.66\pm 0.02$	$0.78\pm 0.06$	$5.60\pm 0.67$
3D-UNet	$\alpha =0.3, \ \beta =0.7, \ \gamma =1$	$0.80\pm 0.06$	$0.77\pm 0.03$	$0.79\pm 0.06$	$4.93\pm 0.78$
nnUNet	$\alpha =0.3, \ \beta =0.7,\ \gamma =1$	$0.84\pm 0.02$	$0.75\pm 0.06$	$0.81\pm 0.08$	$6.33\pm 1.02$
2D-UNet	$\alpha =0.5, \ \beta =0.5,\ \gamma =1$	$0.76\pm 0.03$	$0.67\pm 0.05$	$0.77\pm 0.05$	$5.12\pm 0.98$
3D-UNet	$\alpha =0.5, \ \beta =0.5, \ \gamma =1$	$0.80\pm 0.07$	$0.73\pm 0.09$	$0.81\pm 0.03$	$6.71\pm 1.05$
nnUNet	$\alpha =0.5, \ \beta =0.5,\ \gamma =1$	$0.84\pm 0.05$	$0.74\pm 0.04$	$0.83\pm 0.02$	$6.03\pm 1.30$
2D-UNet	$\alpha =0.7, \ \beta =0.3, \ \gamma =1.5$	$0.79\pm 0.04$	$0.72\pm 0.01$	$0.80\pm 0.04$	$7.01\pm 0.46$
3D-UNet	$\alpha =0.7, \ \beta =0.3, \ \gamma =1.5$	$0.85\pm 0.01$	$0.77\pm 0.02$	$0.82\pm 0.03$	$5.44\pm 1.10$
**nnUNet**	$\alpha =0.7, \ \beta =0.3, \ \gamma =1.5$	$\mathbf {0.89\pm 0.04}$	$\mathbf {0.79\pm 0.07}$	$\mathbf {0.83\pm 0.05}$	$\mathbf {3.52\pm 0.76}$

**Table 4:** Comparison of the automatic segmentation with the manual segmentation from experts as part of the inter-observer variability study (Expert-I vs. Expert-II for the manual segmentation on the PET scans). $\hbox {DSC}_{\text {avg}}$; average Dice Similarity Coefficient, $\hbox {HD}_{\text {mm}}$; Hausdorff Distance (in mm). $\hbox {GT}_{{1ref}}$, Ground truth from Expert-I as the reference. $\hbox {GT}_{{2}}$, Ground truth from Expert-II. CB, Contrast-Based. 12 Bl cases; 12 baseline cases. 12 Fu cases; 12 follow-up cases.

Cases	Automatic Segmentation		Manual GT$_{1ref}$	
	$\hbox {DSC}_{\text {avg}}$	$\hbox {HD}_{\text {diff}}$	$\hbox {DSC}_{\text {avg}}$	$\hbox {HD}_{\text {mm}}$
12 Bl cases				
Manual $\hbox {GT}_{{1ref}}$	$0.89 \pm 0.04$	$3.52 \pm 0.76$	=	=
Manual $\hbox {GT}_{{2}}$	$0.86 \pm 0.06$	$6.10 \pm 1.00$	$0.92 \pm 0.02$	$4.02 \pm 0.21$
CB semi-automatic approach	$0.91 \pm 0.02$	$4.55 \pm 2.03$	$0.90 \pm 0.07$	$4.47 \pm 0.12$
12 Fu cases				
Manual $\hbox {GT}_{{1ref}}$	$0.78 \pm 0.03$	$4.95 \pm 0.12$	=	=
Manual $\hbox {GT}_{{2}}$	$0.80 \pm 0.05$	$4.40 \pm 1.80$	$0.88 \pm 0.01$	$3.97 \pm 1.90$
CB semi-automatic approach	$0.43 \pm 0.11$	$7.40 \pm 3.50$	$0.40 \pm 0.09$	$9.06 \pm 4.22$

**Fig. 7:**
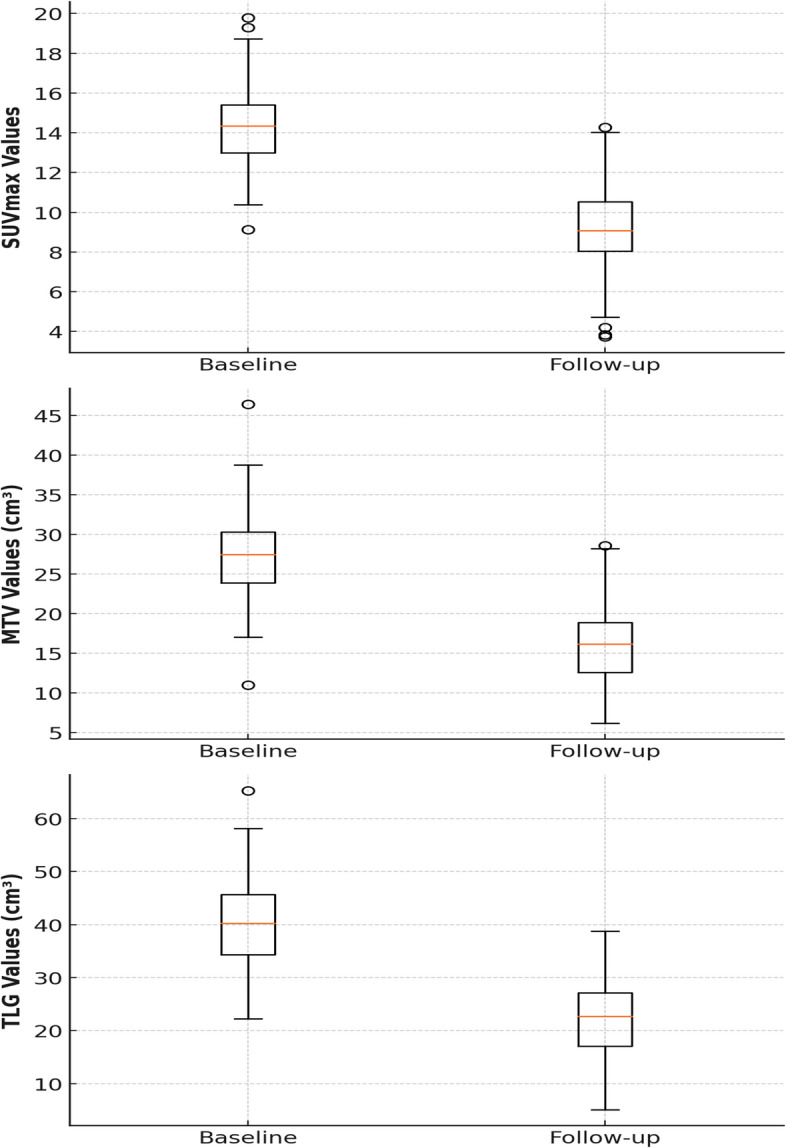
Box plots comparing biomarker values for $\hbox {SUV}_{{max}}$, MTV, and TLG at baseline and follow-up stages for 12 patients. Each box represents the interquartile range (IQR), with the red line inside indicating the median value. Measurements at baseline stage (left) are compared to measurements at follow-up stage (right) for each parameter, illustrating changes after NAC based on the biomarker values extracted from our system.

A comparison of extracted biomarkers was conducted between the predicted masks from our model and the ground truth in the test dataset considering $\hbox {PET}_{{Bl}}$ and $\hbox {PET}_{{FU}}$ scans. For SUV$_{max}$, there is no difference between the manual drawing and our model in SUV$_{max}$ biomarker extraction for $\hbox {PET}_{{Bl}}$ scans. A correlation coefficient of 0.98, with a mean difference of 0.01 ± 0.07, was noted for $\hbox {PET}_{{Fu}}$ scans. Indeed, minor discrepancies were observed due to small holes in the segmented regions. For MTV, the correlation coefficients were 0.95 and 0.90 with a mean difference of 1.57 ± 2.57 cm$^3$ and 0.13 ± 2.97 cm$^3$ for $\hbox {PET}_{{Bl}}$ and $\hbox {PET}_{{FU}}$ scans, respectively. Regarding TLG, the correlation coefficients were 0.91 and 0.87 with mean difference of -6.76 ± 5.65 cm$^3$ and -3.93 ± 6.51 cm$^3$ for $\hbox {PET}_{{Bl}}$ and for $\hbox {PET}_{{FU}}$ scans, respectively. We evaluated the longitudinal variation in key metabolic biomarkers across the 12 cases used to test the models by comparing $\hbox {PET}_{{Bl}}$ and $\hbox {PET}_{{Fu}}$ scans. To quantify these changes, we computed the differences ($\Delta$) for SUV$_{max}$, MTV, and TLG (Fig. 7). The mean difference in SUV$_{max}$ between $\hbox {PET}_{{FU}}$ and $\hbox {PET}_{{Bl}}$ was $-6.02 \pm 1.55$, representing a significant reduction from a baseline mean of 14.00 to 7.98 at follow-up ($p = 0.002$). For MTV, the average reduction was $-9.30 \pm 2.33$ $\hbox {cm}^3$, decreasing from 25.00 $\hbox {cm}^3$ to 15.70 $\hbox {cm}^3$ ($p = 0.007$). Similarly, TLG exhibited a substantial mean decrease of $-14.09 \pm 6.33$, falling from a baseline of 38.00 to 23.91 ($p = 0.010$).

**Table 5:** Comparison of the performance of baseline, fine-tuned, and active learning models in segmentation tasks on the test set of $\hbox {PET}_{{Fu}}$. DSC; Dice similarity coefficient, $\hbox {HD}_{\text {mm}}$; Hausdorff Distance (in mm), IoU; Intersection over Union.

**Methods**	**DSC**	**IoU**	**Sensitivity**	**HD**$_{\text {mm}}$
Baseline model	$0.75 \pm 0.07$	$0.66 \pm 0.09$	$0.76 \pm 0.06$	$5.28 \pm 1.21$
Fine-tuned model	$0.77 \pm 0.04\uparrow$	$0.66 \pm 0.04\downarrow$	$0.77 \pm 0.02\uparrow$	$5.21 \pm 0.55\uparrow$
**Active learning**	$\mathbf {0.78 \pm 0.03}\uparrow$	$\mathbf {0.69 \pm 0.05}\uparrow$	$\mathbf {0.77 \pm 0.04}\uparrow$	$\mathbf {4.95 \pm 0.12}\uparrow$

### Agreement and correlation analysis between expert and proposed method

**Fig. 8:**
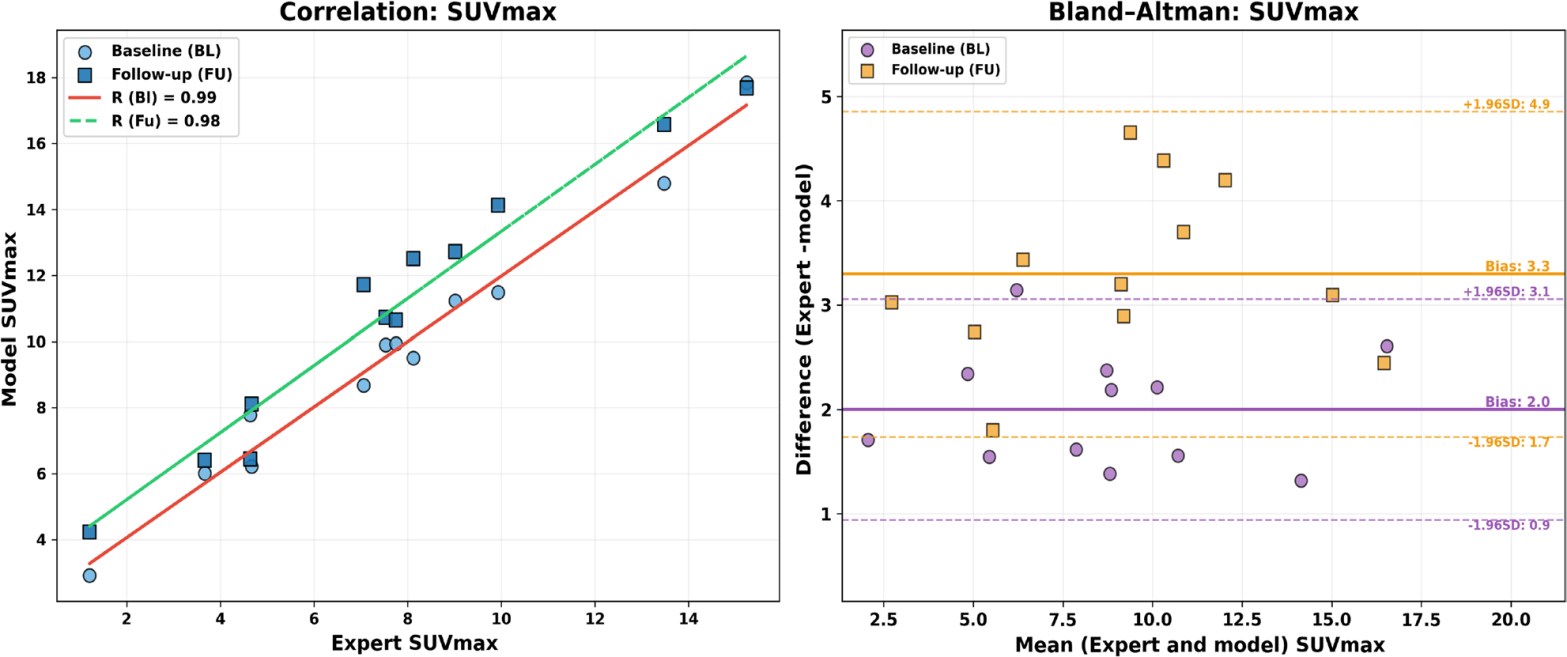
SUV$_{max}$ correlation analysis and agreement study (Bland–Altman plot) between expert and automatic segmentations with nnUNet.

**Fig. 9:**
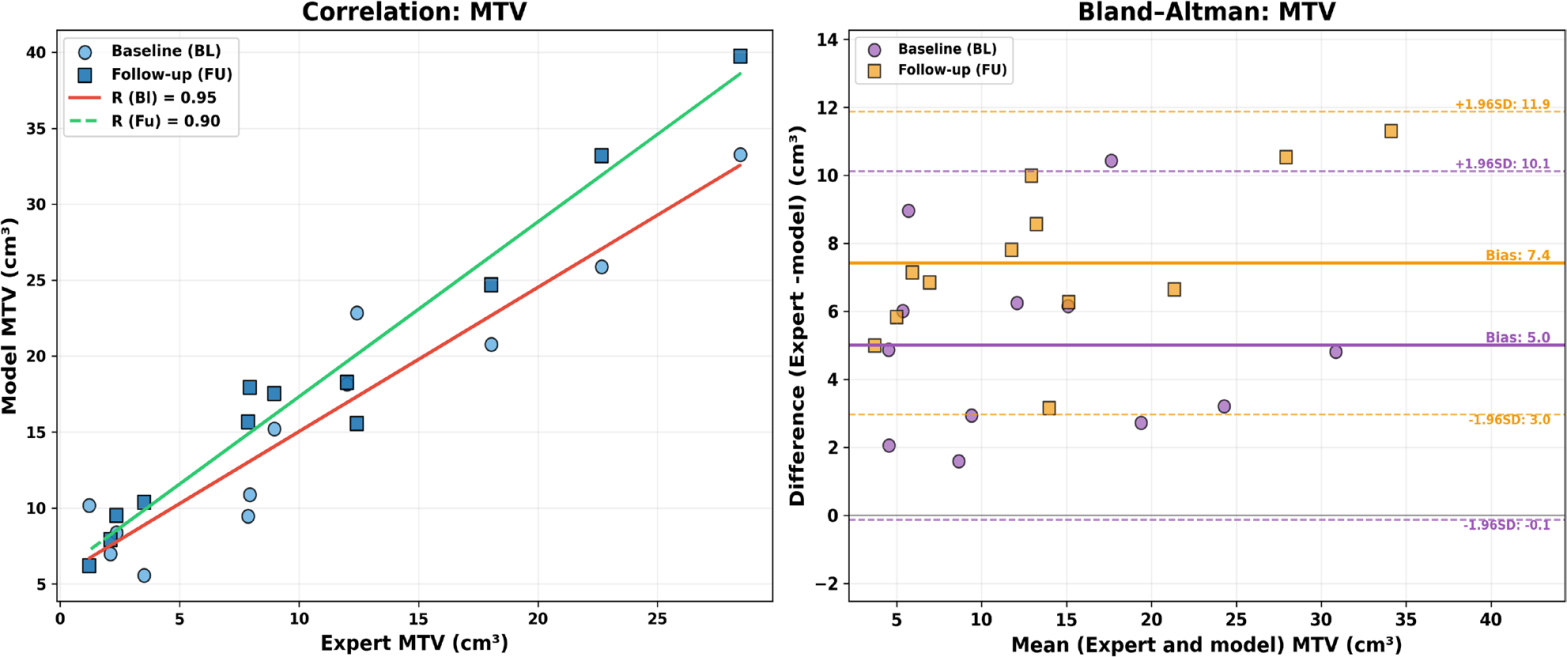
MTV correlation analysis and agreement study (Bland–Altman plot) between expert and automatic segmentations with nnUNet.

**Fig. 10:**
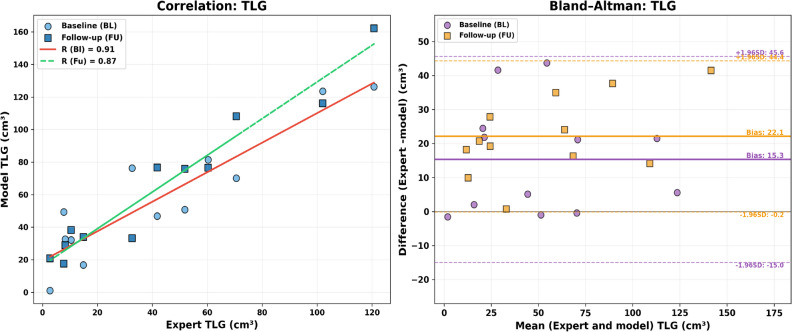
TLG correlation analysis and agreement study (Bland–Altman plot) between expert and automatic segmentations with nnUNet.

For the twelve patients with both expert and nnUNet segmentations, agreement between manual and automatic measurements was assessed using Bland–Altman analysis for $SUV_{max}$, *MTV*, and *TLG*. $SUV_{max}$ showed a mean bias of about 2.00, with narrow limits of agreement and minimal dispersion, reflecting good concordance between expert and automatic segmentations (Fig. 8). At follow-up, a larger positive bias of 3.30 was observed, indicating a modest overestimation while maintaining overall agreement. For *MTV*, a positive mean bias of $5.00\text { cm}^3$ was observed at baseline, indicating a positive bias by the algorithm (Fig. 9). This positive bias was more pronounced at follow-up, with a mean bias of $7.42\text { cm}^3$, although the agreement remained acceptable. For *TLG*, a bias of $15.33\text { cm}^3$ was found at baseline, with slightly higher variability, but no magnitude-dependent bias was observed (Fig. 10). At follow-up, the bias increased to $22.11\text { cm}^3$, reflecting a stronger dispersion compared to baseline. Overall, Bland-Altman analysis demonstrates strong agreement and acceptable biases for all biomarkers. Pearson correlation analysis revealed very high correlation between expert-derived and automatically extracted biomarkers for SUV$_{max}$ ($r = 0.99$) and MTV ($r = 0.95$) for baseline exams, while a strong correlation was observed for TLG ($r = 0.91$). Similarly, analysis on follow-up cases showed high correlations for SUV$_{max}$ ($r = 0.98$), MTV ($r = 0.90$), and TLG ($r = 0.87$).

**Fig. 11:**
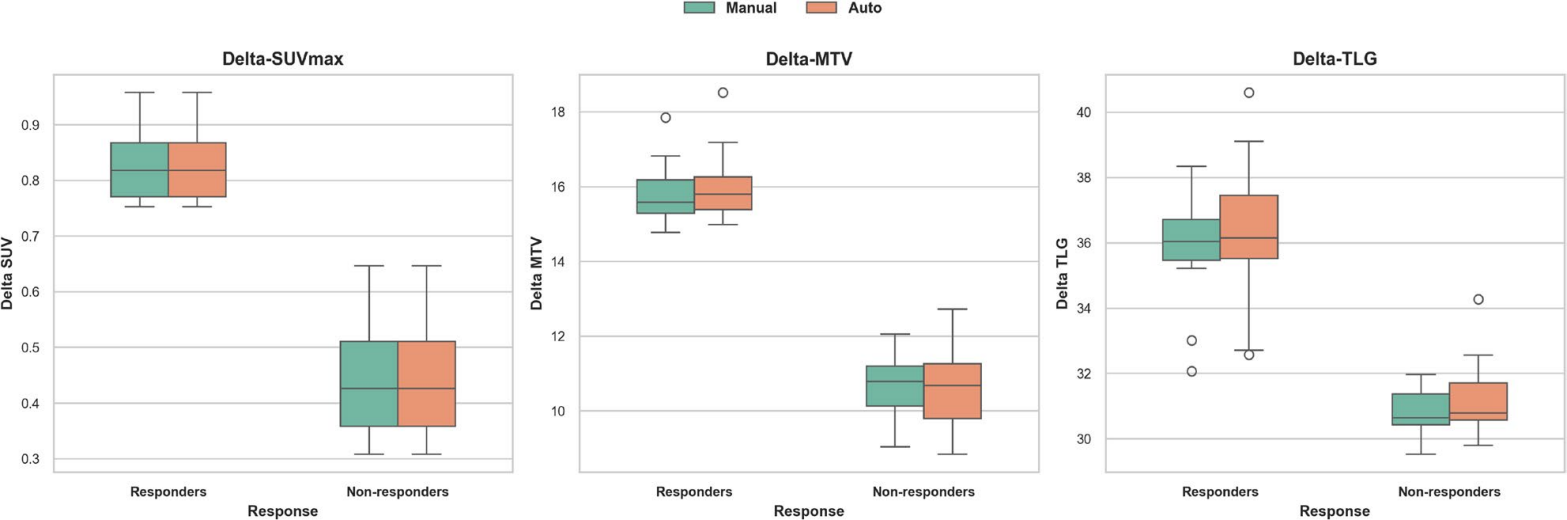
Comparison of $\Delta \textrm{SUV}_{\max }$, $\Delta \textrm{MTV}$, and $\Delta \textrm{TLG}$ between responders and non-responders using manual and automatic segmentations.

Finally, longitudinal analysis showed that changes in SUV$_{max}$, $\textrm{MTV}$, and $\textrm{TLG}$ derived from nnUNet segmentations closely matched those obtained from expert contours. Moreover, changes in SUV$_{max}$, $\textrm{MTV}$, and $\textrm{TLG}$ exhibited consistent patterns between responders and non-responders, regardless of whether they were extracted using manual or automatic segmentation methods. Figure 11 illustrates the distribution of these changes, confirming differences in $\Delta \textrm{SUV}_{\max }$, $\Delta \textrm{MTV}$, and $\Delta \textrm{TLG}$ between responders and non-responders, using both manual and automatic segmentation methods. For $\Delta \text {SUV}_{\max }$, responders demonstrated significantly higher metabolic reductions compared with non-responders from manual segmentation ($p = 0.015$), or automatic segmentation ($p = 0.015$). For $\textrm{MTV}$, the magnitude of change was higher in responders than in non-responders for both manual ($p = 0.013$) and automatic segmentation ($p = 0.011$) methods. Similarly, for $\textrm{TLG}$, responders showed greater changes compared with non-responders from manual segmentation ($p = 0.027$), or from automatic segmentation ($p = 0.020$). These findings indicate clear treatment-related shifts in all three biomarkers between responders and non-responders, regardless of the segmentation approaches. Then automatic segmentation does not substantially affect the interpretation of treatment response and supports its reliability for longitudinal PET biomarker assessment in the neoadjuvant chemotherapy setting.

## Discussion

The proposed deep learning framework demonstrates strong performance in breast cancer lesion segmentation and PET-derived biomarker extraction. Among the evaluated architectures, nnUNet consistently achieved the highest segmentation accuracy, outperforming both 2D UNet and MONAI-based 3D UNet in terms of Dice Similarity Coefficient, Intersection over Union, and Hausdorff Distance. On baseline $\hbox {PET}_{{Bl}}$ scans, the optimized nnUNet configuration achieved a Hausdorff distance of 3.52 ± 0.76 mm, while its Dice performance approached inter-observer agreement levels (manual DSC 0.92 ± 0.02). This indicates that the automated model operates within expert variability and provides boundary delineation comparable to manual annotation. The framework was trained and validated on 231 PET scans, ensuring statistical robustness and reliable performance estimation. As expected, the highest performance was observed on $\hbox {PET}_{{Bl}}$ scans, since the model was initially trained on this dataset. In contrast, segmentation on follow-up $\hbox {PET}_{{Fu}}$ scans was inherently more challenging due to reduced tumor uptake after neoadjuvant chemotherapy, leading to lower lesion-to-background contrast. Despite this increased difficulty, nnUNet maintained stable and clinically acceptable performance on $\hbox {PET}_{{Fu}}$ scans. Importantly, model refinement through fine-tuning and active learning strategies led to a 3% improvement in $\hbox {PET}_{{Fu}}$ segmentation accuracy. By incorporating challenging low-uptake cases during retraining, the network adapted more effectively to subtle metabolic patterns and heterogeneous tumor presentations. This demonstrates the adaptability of the proposed framework and highlights its potential for longitudinal monitoring applications.

When compared to semi-automatic contrast-based segmentation methods, the proposed fully automatic system achieved comparable performance on $\hbox {PET}_{{Bl}}$ scans (DSC 0.89 ± 0.04 versus 0.90 ± 0.07). However, the automatic approach provided improved boundary consistency and reduced inter-case variability. On $\hbox {PET}_{{Fu}}$ scans, where lesion contrast is substantially reduced, the semi-automatic method showed instability and limited reliability (Table 4), whereas the proposed framework maintained a Dice score of 0.78 ± 0.03. This highlights the advantage of data-driven volumetric learning in challenging low-intensity scenarios. Beyond geometric accuracy, a key strength of the proposed framework lies in its end-to-end design. The system automatically localizes the region of interest, performs volumetric tumor segmentation, and directly extracts quantitative biomarkers including $\hbox {SUV}_{{max}}$, MTV, and TLG. This fully integrated pipeline eliminates multiple manual processing steps and ensures reproducible biomarker computation. Quantitative analysis further supports the reliability of the automated method. In particular, correlation study confirmed the consistency between expert-derived and nnUNet-derived biomarkers. Pearson correlation coefficients revealed a very strong agreement for $\hbox {SUV}_{{max}}$ ($r = 0.99$) and MTV ($r = 0.95$), alongside a strong correlation for TLG ($r = 0.91$). These high values indicate that inter-patient metabolic variability was well-preserved by the automated framework. Bland–Altman analysis demonstrated minimal bias and no evidence of proportional error, confirming equivalence between automatic and expert measurements. These results demonstrate that the proposed nnUNet-based framework provides expert-level segmentation accuracy and reliable biomarker extraction within a fully automated workflow. By reducing clinician workload, minimizing subjective variability, and accelerating analysis time, the method supports standardized and reproducible tumor assessment in routine clinical practice while maintaining performance comparable to manual or semi-automatic approaches.

Regarding these segmentation results, our method demonstrates performance comparable to previous studies on lesion segmentation using PET images^41–44^. Our segmentation performance is comparable to that reported by Moreau et al.^41^, who achieved a mean DSC of $0.78 \pm 0.07$ for $\hbox {PET}_{{Bl}}$ scans and $0.56 \pm 0.11$ for $\hbox {PET}_{{Fu}}$ scans using a UNet network to segment metastatic breast cancer lesions on 10 patients (each with one baseline and one follow-up exam). Our model presents overall better results, particularly on $\hbox {PET}_{{Fu}}$ scans. However, the segmentation of metastatic lesions poses further challenges due to their variable sizes and locations, which may reduce the overall performance of the proposed method. Vogl et al.^45^ explored a data-driven machine learning approach for a Computer-Aided Diagnosis system using dynamic contrast-enhanced-MRI, diffusion-weighted imaging, and $^{18}$F-FDG PET. They employed a random forest classifier combined with multi-parametric PET/MRI intensity-based features for breast lesion segmentation, achieving a DSC of $0.67 \pm 0.23$. They employed a leave-one-out cross-validation approach to evaluate lesion segmentation performance on a dataset of 34 cases. A study conducted by Qiao et al.^46^ on breast tumor segmentation in PET imaging through attentive transformation-based normalization yielded promising results, achieving a DSC of $0.86 \pm 0.06$. They conducted their evaluation using a subset of 10 scans for testing, selected from a total of 54 scans in their dataset. Building upon their findings, our research endeavors to extend the boundaries of tumor segmentation by integrating advanced deep learning and combined loss function techniques.

**Fig. 12:**
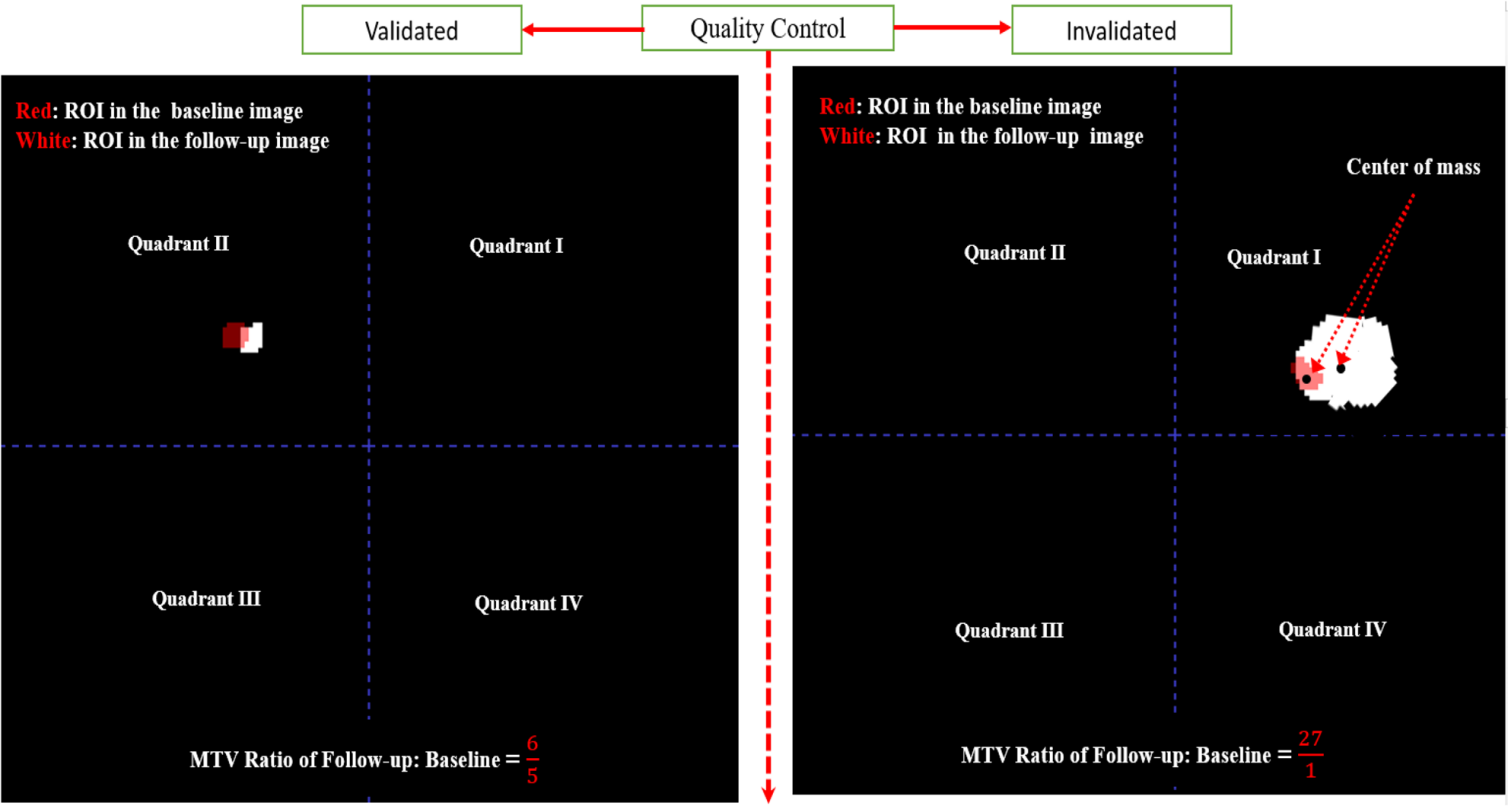
Example of a quality control method from predicted tumor areas from $\hbox {PET}_{{Bl}}$ and $\hbox {PET}_{{Fu}}$ from the same patient. In the Validated scenario on the left, both ROIs automatically predicted are located in the same quadrant, with the MTV ratio between them being below the specified threshold. In the Invalidated scenario on the right, although the ROIs are in the same quadrant, the MTV ratio exceeds the threshold. This is one of the outliers identified by our quality control system.

By accurately segmenting the breast lesions at baseline and follow up stages, we were able to extract relevant biomarkers from the segmented regions to track breast cancer progression after the first course of NAC. Several research studies have investigated biomarkers to predict the response to NAC in primary breast cancer. Incorporating additional markers like TLG and $\hbox {SUV}_{{max}}$ could enhance the accuracy of pCR prediction^47^. In a study of Humbert et al.^48^, the value of metabolic tumor response, assessed using $^{18}$F-FDG-PET, was evaluated for its ability to predict the pathological complete response in women with triple-negative breast cancer. The study found that combining a low metabolic response ($\Delta$ $\hbox {SUV}_{{max}}$ $\le$ -50%) with positive epidermal growth factor receptor status predicted non-pCR with 92% of accuracy. Furthermore, Humbert et al.^14^ demonstrated that low tumor metabolism after the first cycle of NAC ($\hbox {SUV}_{{max}}$ < 2.1) strongly predicts pCR in HER2-positive breast cancer, suggesting its potential as an early indicator of treatment success. We evaluated the longitudinal variation in key metabolic biomarkers across the 12 cases used for testing by comparing $\hbox {PET}_{{Bl}}$ and $\hbox {PET}_{{Fu}}$ scans. There is a notable reduction in metabolic, volumetric, and total lesion burden parameters (Fig. 7). The mean difference in $\hbox {SUV}_{{max}}$ was $-6.02 \pm 1.55$, representing a $43.0\%$ reduction from a baseline mean of 14.00 to 7.98 at follow-up ($p = 0.002$). For MTV, the average reduction was $-9.30 \pm 2.33$ $\hbox {cm}^3$, reflecting a $37.2\%$ decrease from 25.00 $\hbox {cm}^3$ at baseline to 15.70 $\hbox {cm}^3$ at follow-up ($p = 0.007$). Similarly, TLG exhibited the most substantial decrease with a mean reduction of $-14.09 \pm 6.33$, falling from a baseline of 38.00 to 23.91 ($37.1\%$ reduction; $p = 0.010$). These observed variations reinforce the utility of PET-derived quantitative biomarkers as indicators of therapeutic efficacy, supporting their integration into clinical decision-making for personalized treatment strategies. Han et al.^7^ investigated the percentage change in $\hbox {SUV}_{{max}}$ (%$\Delta$ $\hbox {SUV}_{{max}}$) and highlighted its role as a widely studied parameter for distinguishing metabolic responders from non-responders in interim or post-treatment PET scans. Li et al.^49^ explored variations in biomarkers to predict pCR for each breast cancer subtype group and found that biomarker variation was in 20 out of 72 (27.8%) luminal cases, 46 out of 79 (58.2%) HER2-positive cases, and 37 out of 78 (47.4%) triple-negative breast cancer (TNBC) cases. In our study, we analyzed the variation of SUV$_{max}$ for each breast cancer subtype cases. For the luminal subtype, the reduction was from $8.92 \pm 2.33$ at baseline stage to $6.96 \pm 1.98$ at follow-up stage (21.96% of decrease). For the HER2 subtype, the reduction was from $15.08 \pm 4.17$ at baseline stage to $9.75 \pm 0.13$ at follow-up stage (35.34% of decrease). For the TNBC subtype, the reduction was from $19.08 \pm 7.11$ at baseline stage to $10.71 \pm 9.80$ at follow-up stage (42.70% of decrease).

Regular monitoring and integration of clinical and imaging data are crucial for comprehensive evaluation and informed decision-making. In breast cancer patients undergoing NAC, a pCR can be accurately predicted by a reduction in $^{18}$F-FDG PET uptake after just one course of chemotherapy^8^. Berriolo-Riedinger et al. also demonstrated that a relative decrease of 60% in SUV$_{max}$ or 50% in SUV$_{average}$ serves as a predictive threshold for achieving pCR. These findings could participate to the patient management by identifying ineffective chemotherapy early or supporting decisions to proceed with dose-intensive preoperative chemotherapy in patients who are responding. Végran et al.^50^ explored the potential of microarray analyses to identify markers, such as genes, associated with pCR. Guiu et al.^51^ showed also that pCR serves as a prognostic factor, particularly in patients with hormone receptor-positive tumors. Payan et al.^52^ combined clinical, histopathological, blood flow (from PET scans), and metabolic features (from the same PET scans), including image texture information, at baseline phase to predict pCR in breast cancer subgroups. Payan et al. found that combining metabolic features, texture features, and clinical data improved pCR prediction, but adding blood flow information did not modify the results. Recent state-of-the-art approaches have begun integrating imaging biomarkers with non-imaging clinical data to enhance prognostic accuracy^53,54^. For instance, while our framework focuses on the precise extraction of PET-derived biomarkers like $\Delta SUV_{max}$ and $\Delta TLG$, recent ensemble methods have demonstrated that combining such imaging features with clinical measures creates a synergy that significantly improves the prediction of long-term patient outcomes, such as longitudinal motor scores in neurodegenerative diseases. Furthermore, the challenges we observed in segmenting low-uptake lesions in $\hbox {PET}_{{Fu}}$ scans could potentially be mitigated by emerging semi-supervised transfer learning (SSTL) techniques. These approaches leverage limited annotations and radiomics for tumor segmentation. They enable robust generalization across tracers and metabolic intensities, reinforcing the clinical value of automated pipelines for monitoring disease progression and treatment response.

However, our work has certain limitations. The model was trained using PET images only, without leveraging simultaneously acquired CT information that could further improve segmentation robustness. In addition, validation was conducted on a single-center dataset, and external multi-center evaluation is required to confirm robustness across different scanners and acquisition protocols. Then, future work should explore multimodal integration, larger validation cohorts, and incorporation of radiomic and clinical features for predictive modeling.

## Conclusion

In conclusion, this study presents an approach for the automatic segmentation and biomarker computation of breast cancer lesions from $^{18}$F-FDG PET scans. The proposed pipeline is based on deep learning architectures combined with a weighted loss function. It achieved consistent segmentation performance at baseline, prior to NAC, as well as at the early follow up after one treatment cycle. The framework incorporates fine tuning strategies and elements of active learning, together with quality control procedures to support segmentation reliability. This approach may contribute to more efficient and reproducible assessment of disease evolution during treatment, and could support clinicians in treatment monitoring and decision making. In future work, integration of imaging biomarkers with clinical and pathological variables may allow the development of predictive models for treatment response and survival outcomes. The developed tool could serve as a basis for further development toward a computer aided detection system within the breast cancer management workflow. The source code for this project is publicly available on GitHub at https://github.com/Tareke12Tewele12?tab=repositories.

## Data Availability

The datasets used and analysed during the current study are not publicly available but are available from the corresponding author on reasonable request. The source code implementation for this study is publicly accessible on GitHub at https://github.com/Tareke12Tewele12?tab=repositories

## Abbreviations

BCE: Binary Cross-Entropy
CT: Computed Tomography
DSC: Dice Similarity Coefficient
FDG: Fluorodeoxyglucose
FN: False Negative
FP: False Positive
FTL: Focal Tversky Loss
GT: Ground Truth
HD: Hausdorff Distance
IoU: Intersection over Union
$$\hbox {Model}_{{BL}}$$: Baseline Model
MRI: Magnetic Resonance Imaging
MTV: Metabolic Tumor Volume
NAC: Neoadjuvant Chemotherapy
NETs: Neuroendocrine Tumors
nnUNet: No-New-Net
pCR: Pathological Complete Response
PET: Positron Emission Tomography
$\hbox {PET}_{{BL}}$: Positron Emission Tomography before first course of NAC (baseline)
$\hbox {PET}_{{FU}}$: Positron Emission Tomography after first course of NAC (follow-up)
ROI: Region of Interest
SUV: Standardized Uptake Value
$\hbox {SUV}_{{max}}$: Maximum Standardized Uptake Value
SSTL: Semi-Supervised Transfer Learning
TI: Tversky Index
TN: True Negative
TP: True Positive
